# Copy number variation profiling in pharmacogenetics *CYP-450* and *GST* genes in Colombian population

**DOI:** 10.1186/s12920-019-0556-x

**Published:** 2019-07-19

**Authors:** Brian Ramírez, María José Niño-Orrego, Daniel Cárdenas, Kevin Enrique Ariza, Karol Quintero, Nora Constanza Contreras Bravo, Caroll Tamayo-Agudelo, María Alejandra González, Paul Laissue, Dora Janeth Fonseca Mendoza

**Affiliations:** GENIUROS Research Group, Center For Research in Genetics and Genomics – CIGGUR, School of Medicine and Health Sciences, Universidad Del Rosario, Carrera 24 N° 63C-69, CP 112111, Bogotá DC, Colombia

**Keywords:** DNA copy number variation, Allele frequency, Personalized medicine, Pharmacogenomics,DNA

## Abstract

**Background:**

Copy Number variation (CNVs) in genes related to drug absorption, distribution, metabolism and excretion (ADME) are relevant in the interindividual variability of drug response. Studies of the CNVs in ADME genes in Latin America population are lacking. The objective of the study was to identify the genetic variability of CNVs in *CYP-450* and *GST* genes in a subgroup of individuals of Colombian origin.

**Methods:**

Genomic DNA was isolated from 123 healthy individuals from a Colombian population. Multiplex Ligation-Dependent Probe Amplification (MLPA) was performed for the identification of CNVs in 40 genomic regions of 11 *CYP-450* and 3 *GST* genes. The genetic variability, allelic and genotypic frequencies were analyzed.

**Results:**

We found that 13 out of 14 genes had CNVs: 5 (35.7%) exhibited deletions and duplications, while 8 (57.1%) presented either deletions or duplications.. 33.3% of individuals carried deletions and duplications while 49.6% had a unique type of CNV (deletion or duplication). The allelic frequencies of the *CYP* and *GST* genes were 0 to 47.6% (allele null), 0 to 17.5% (duplicated alleles) and 37 to 100% (normal alleles).

**Conclusions:**

Our results describe, for the first time, the genomic profile of CNVs in a subgroup of Colombian population in *GST* and *CYP-450* genes. *GST* genes indicated greater genetic variability than CYP-450 genes. The data obtained contributes to the knowledge of genetic profiles in Latin American subgroups. Although the clinical relevance of CNVs has not been fully established, it is a valuable source of pharmacogenetic variability data with potential involvement in the response to medications.

## Background

Interindividual variability response to drugs has been associated with multiple genetic and environmental factors [1]. Genetic variants in genes encoding proteins related to drug absorption, distribution, metabolism and excretion (ADME) have shown to impact on pharmacokinetics, pharmacodynamics efficacy and safety [2–4]. In view of the variation of pharmacogenes relevant in clinical practice, the FDA (US Food and Drug Administration) and EMA (European Medicines Agency) have recognized the benefit of genotyping some validated biomarkers for the identification of cases at risk of potential toxicity or therapeutic failure. In this context, genetic analysis facilitates the selection of a safer and more effective pharmacological management for each patient.

Despite the fact that Single Nucleotide Variants (SNVs) are the most widely studied variants, there has been a recent recognition in the influence of CNVs on interindividual differences in drug medication response [5]. It has been estimated that around 12% of the human genome contains CNVs, which are defined as duplications or deletions of DNA segments from 1 Kb to 3 Kb [1, 6]. It has been determined that several pharmacogenes of clinical relevance (e.g. *CYP2D6*, *GSTT1*, *GSTM1*, *SULT1A1*, *CYP2A6*, and *UGT2B17*) contain CNVs associated to the variation of enzymatic activity observed among different populations*. CYP2D6* and *CYP2A6* constitute coding genes for Phase I metabolism enzymes and display the greatest number of reported CNVs [4]. Regarding the Phase II metabolism enzymes, CNVs in the glutathione transferase enzymes and sulfotransferases have been reported. These genes are involved in drug metabolism and detoxification of xenobiotics [1, 7–9]. In the Latin-American population there is a notable absence of genetic studies, and with exception of CYP2D6 there is a gap regarding frequency of drug-related CNVs [10]. Analyses using autosomal and sexual markers performed in the Latin American population have indicated a great variation in the influence of African / European and Native ancestry between individuals and geographic regions. The analysis of SNPs in more than 6000 individuals in 5 Latin American countries has estimated that the highest proportion of African ancestry occurs in Brazil (9.3%) and Colombia (9.6%) (with ranges for other countries between 4.6 and 9.6); the native in Peru (64.8%) (ranges of 12.1 to 64.8%) and the European in Brazil (78.6%) and Colombia (61.2%) (ranges of 30.6 to78.6%) [11]. These findings reflect a high heterogeneity in the structure of these populations [10, 11].

Although clinical relevance still needs to be established, CNVs play a clear role in drug-related genes as they alter metabolism and therapeutic response [1, 4, 12, 13].

The present study analyzed 40 genomic regions of *GSTM1, GSTP1, GSTT1, CYP1A1, CYP1A2, CYP1B1, CYP2A6, CYP2B6, CYP2C9, CYP1C19, CYP2D6, CYP2E1, CYP3A4,* and *CYP3A5* genes through Multiplex Ligation-Dependent Probe Amplification (MLPA) in 123 healthy individuals from a cohort belonging to the Colombian population. Our results indicated that 13 out of 14 genes exhibited CNVs defined by the presence of deletions and/or duplications in at least one exon. 33.3% of the genes presented the combination of both. Our population exhibited variability in CNVs: 50% of individuals carried deletions and duplications while 39% had a unique type of CNV (deletion or duplication). According to the number of *CYP-450* or *GST* active copies, the individuals can be potentially defined as poor metabolizers (PM) or ultrarapid metabolizers (UM) [14]. We identified that 83% of the analyzed individuals presented CNVs in one or several of the CYP-450 and / or GST genes studied.

Our results constitute the first description of the frequency of CNVs in a Colombian cohort, contributing to the knowledge of these CNVs in the Latin American population and their potential utilization in the clinical setting.

## Methods

### Study population

Peripheral blood samples were obtained for DNA extraction from 123 healthy donors from the Center For Research in Genetics and Genomics (Bogotá, Colombia). More precisely, detailed information on the methodology for healthy individuals’ enrollment was included in the internet site of the institution. Each participant was informed with respect to: project objectives, sampling procedure, risks and results management. All the individuals signed an informed consent regarding the use of their DNA for research. 58% of the participants were women and 42% men, with ages ranging from 20 to 59 years. All the chosen subjects were born in Bogota, the capital of Colombia, a city with an estimated population structure with a predominance of native ancestry (52%), followed by European and African (45 and 3% respectively). None of the participants were asked for their self-reported ethnicity, and ancestry was assumed as indicated in previous studies based on the analysis of AIM’s in individuals from this same population. [15] . The sample size was calculated considering the estimation of a proportion with a confidence level of 95% (α: 0.05, z: 1.96), p (sample proportion) 3% and e (margin of error) 3% [16]. Considering that this is the first study that analyzes genomic regions in 14 *CYP-450* and *GST* genes by MLPA in the Colombian population, the value of sample proportion (p) was estimated according to the frequency of alleles with duplication/deletion of the *CYP2D6* gene identified by Isaza et al. [17]. The sample size (with finite population correction and) was equal to 125.

The experimental procedures of this study were approved by the Ethics Committee of Universidad del Rosario (CEI-AMH002–000174). The study was conducted according to the principles of the Helsinki Declaration (institutional review board reference CS/ABN062).

### Multiplex ligation-dependent probe amplification (MLPA)

Genomic DNA was isolated from blood samples using the Salting-out method. MLPA was performed using the commercial kit SALSA MLPA P128-C1 Cytochrome P450 probe mix (#P128-C1, MRC-Holland, Amsterdam) according to the manufacturer’s instructions. As stated by the information from the kit, the P128-C1 Cytochrome P450 probemix contains 52 MLPA probes with amplified products between 128 and 504 nt. Additionally it includes 4 DNA quantity fragments (Q-fragments), three DNA denaturation control (D.-fragments), an X-fragment and one Y-fragment (https://www.mlpa.com). For the identification of the CNVs, 40 genomic regions in 14 CYP-450 and GST genes, which were contained in the commercial kit, were used in this analysis (Table 1).

**Table 1:** Genomic Regions analyzed

	Exon1	Exon2	Exon3	Exon4	Exon5	Exon6	Exon7	Exon8	Exon9	Exon10	Exon13	Downstream Exon9
*CYP1A1*	✔	✔	✔									
*CYP1A2*		✔		✔			✔					
*CYP1B1*	✔		✔									
*CYP2A6*	✔	✔	✔		✔							
*CYP2B6*		✔	✔	✔								
*CYP2C19*		✔				✔			✔			
*CYP2C9*	✔						✔	✔	✔			
*CYP2D6*	✔				✔	✔						✔
*CYP2E1*					✔	✔		✔				
*CYP3A4*	✔					✔					✔	
*CYP3A5*		✔		✔						✔		
*GSTM1*			✔		✔							
*GSTP1*			✔	✔								
*GSTT1*	✔											

Each gene was analyzed with at least two probes, with the exception of *GSTT1*, which was determined by one probe in the exon 1. The genes of the cytochrome P450 and Glutathione S-transferase included in the analysis were: *GSTM1, GSTP1, GSTT1, CYP1A1, CYP1A2, CYP1B1, CYP2A6, CYP2B6, CYP2C9, CYP2C19, CYP2D6, CYP2E1, CYP3A4,* and *CYP3A5*.

For each MLPA reaction, 50 ng of DNA of each sample was denatured in a thermocycler for 5 min at 98 °C. After cooling to 25 °C, the probemix and the MLPA buffer were added to each sample, mixed and incubated for 1 min at 95 °C followed by 16 h of hybridization at 60 °C. The ligation reaction was performed incubating at 54 °C the ligase-65 mix, followed by heating at 98 °C for 5 min. Thereafter PCR was performed using exon-specific probes with universal-tagged primers. The PCR consisted of 35 amplification cycles, (95 °C for 30 s, 60 °C for 30 s and 72 °C for 1 min), followed by a 20-min incubation at 72 °C. The amplified products were separated by capillary gel electrophoresis in an Applied Biosystems 3500 Genetic Analyzer using GeneScan350 ROX as standard internal lane size.

### Data analysis

The analysis of MLPA was performed using coffalyser.Net software (https://www.mlpa.com). Data generated by SALSA MLPA P128-C1 Cytochrome P450 probe mix was normalized intra-sample (within each sample, compare each probe peak to the peaks of the reference probes). The relative probe signals determined are then used in intersample normalisation (final probe ratios are determined by comparing the relative probe peak in the DNA sample of interest to all reference samples.). The quality control and data normalization were performed using reference probes (SALSA MLPA P128-C1 Cytochrome P450 probe mix).

CNV status was assigned as follows: if a deletion or duplication on either of the exons in the gene was detected, the whole gene was categorized as deleted or duplicated respectively. The copy number was determined in accordance with instructions of SALSA MLPA P128-C1 Cytochrome P450 probe mix. The relationship between copy number status and the typical distribution of Dosage Quotient Distribution (DQs) (based on a large number of samples at MRC-Holland) was: DQ = 0 (homozygous deletion); 0.40 < DQ < 0.65 (heterozygous deletion); 0.80 < DQ < 1.20 (Normal); 1.30 < DQ < 1.65 (heterozygous duplication); 1.75 < DQ < 2.15 (homozygous duplication), all other values (ambiguous result) (www.mlpa.com).

All the samples were divided into 14 categories based on the genotype combination of the 14 genes. Moreover, we determined the frequency of the individuals for each category. Likewise, individuals were categorized into 4 defined groups: if they had only deletions in one or more genes, only duplications, deletions and duplications or no CNVs. The analysis for the allelic and genotypic frequencies for each gene was determined using SNPStats (https://www.SNPstats.net/start.htm).

## Results

**Fig. 1:**
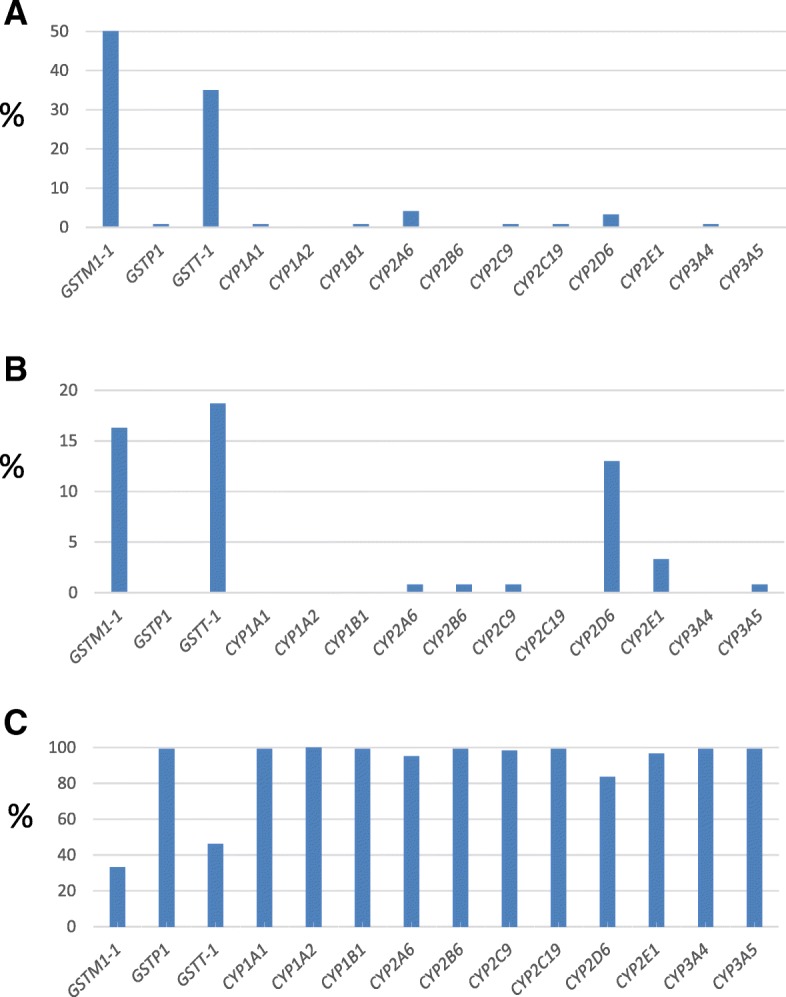
Copy number variation in CYP-450 and GST genes. **a** Deletions, **b** Duplications, **c** No CNVs

CNVs were analyzed using a panel of MLPA with 11 genes from the family of cytochrome P-450 (*CYP1A1, CYP1A2, CYP1B1, CYP2A6, CYP2B6, CYP2C9, CYP2C19, CYP2D6, CYP2E1, CYP3A4, CYP3A5*) and 3 from the glutathione S- Transferase family (*GSTM1, GSTP1, GSTT1*). In 92.9% of the genes, there were duplications and/or deletions identified, *CYP1A2* was the only gene with no CNVs identified. The frequency of deletions and duplications were 0 to 50.4% and 0 to 18.7% respectively (Fig. 1). Our study revealed that CNVs were frequent in the glutathione S- transferase genes, *GSTM1* showed a percentage of individuals with deletion-duplication of 67%, followed by *GSST1* with 54%. In *CYP-450* genes, *CYP2D6* was the most polymorphic (13% duplication and 3.3% deletion). Thirteen out of 14 genes have some CNVs: 5 (35.7%) exhibited deletions and duplications while and 8 (57.1%) only deletions or duplications.

**Fig. 2:**
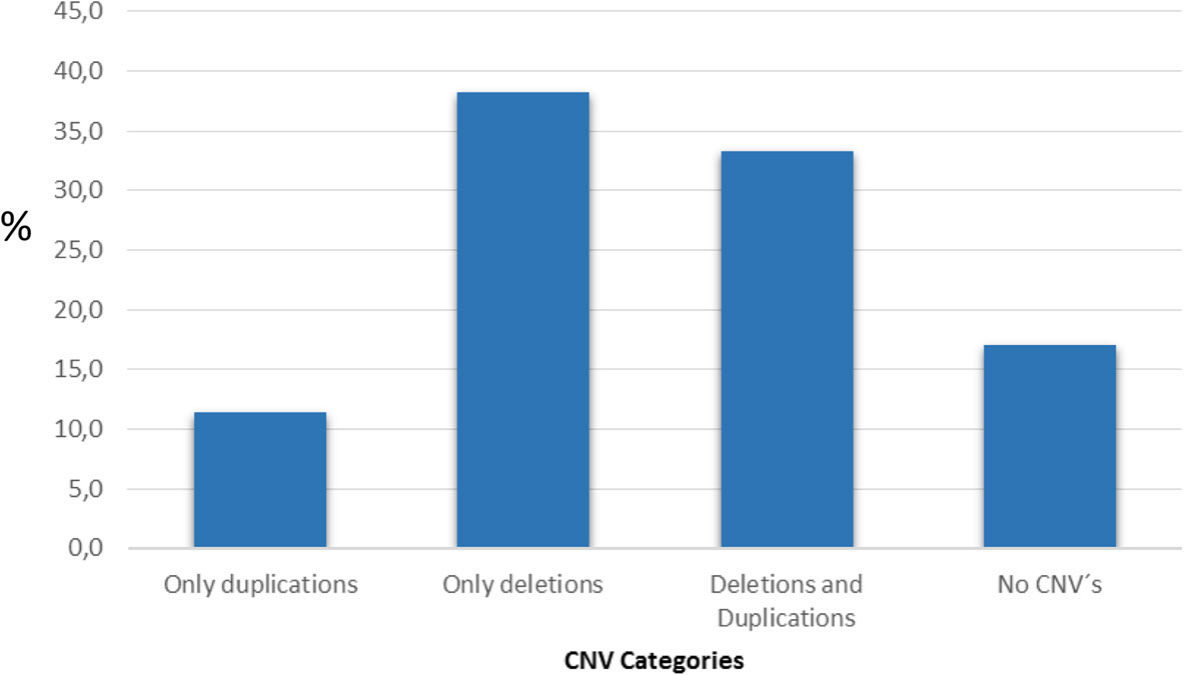
Distribution of CNVs in analyzed genes

The samples were distributed into 14 categories based on the mutational status of the 14 genes analyzed: 1) wild-type for all genes; 2) homozygous deletion for one or more genes; 3) heterozygous deletion for one or more genes; 4) homo or heterozygous deletion for one or more genes; 5) homozygous duplication for one or more genes; 6) heterozygous duplication for one or more genes; 7) hetero and homozygous deletions; 8) heterozygous duplications/homozygous deletions; 9) homozygous duplications/heterozygous deletions; 10) homozygous duplications/ homozygous deletions; 11) homozygous duplications/heterozygous duplications; 12) heterozygous duplications/homo y heterozygous deletions; 13) hetero and homozygous duplications/heterozygous deletions and 14) hetero and homozygous duplications/homozygous deletions (Table 2)In categories 1, 2 and 10 we observed the greatest number of individuals (17, 23, and 12% respectively). 35% of subjects were carries of different combinations of CNVs (category 7–14). When categorizing the individuals according to the type of CNV, it was possible to establish that most of them were carriers of only deletions (38%), while the presentation of exclusive duplications was only evidenced in 11% of the population. The combination of CNVs was identified in 33% of the cases and no CNV were identified in 17% (Fig. 2).

**Table 2:** Population categorization by mutational status

Category	Mutational Status	Percentage (%)
1.	Wild type	17
2.	Homozygous deletions	23
3.	Heterozygous deletions	7
4.	Homo and heterozygous deletions	9
5.	Homozygous duplications	9
6.	Heterozygous duplications	1
7.	Hetero and homozygous deletions	2
8.	Heterozygous duplications and homozygous deletions	10
9.	Homozygous duplications and heterozygous deletions	5
10.	Homozygous duplications and homozygous deletions	12
11.	Homozygous duplications and heterozygous duplications	2
12.	Heterozygous duplications, homozygous and heterozygous deletions	2
13.	Heterozygous deletions, Heterozygous and homozygous duplications	2
14.	Hetero and homozygous duplications and homozygous deletions	1

The genotypic frequencies established for each gene are described in Table 3. The allelic frequencies of the *CYP* and *GST* studied genes were determined with ranges for alleles deleted from 0 to 47.6%, alleles duplicated from 0 to 17.5% and normal alleles from 37 to 100% (Table 4).

**Table 3:** Genotypic frequencies

	*GSTM1*	*GSTP1*	*GSTT1*	*CYP1A1*	*CYP1A2*	*CYP1B1*	*CYP2A6*	*CYP2B6*	*CYP2C9*	*CY2C19*	*CYP2D6*	*CYP2E1*	*CYP3A4*	*CYP3A5*
Del/Wt	5.7	0.8	15.4	0.8	0	0.8	4.1	0	0.8	0.8	3.3	0	0.8	0
Del/Del	44.7	0	19.5	0	0	0	0	0	0	0	0	0	0	0
Dup/Wt	1.6	0	2.4	0	0	0	0.8	0.8	0.8	0	10.6	3.3	0	0.8
Dup/Dup	14.6	0	16.3	0	0	0	0	0	0	0	2.4	0	0	0
Wt/Wt	33.3	99.2	46.3	99.2	100	99.2	95.1	99.2	98.4	99.2	83.7	96.7	99.2	99.2

**Table 4:** Allelic frequencies

	*GSTM1*	*GSTP1*	*GSTT1*	*CYP1A1*	*CYP1A2*	*CYP1B1*	*CYP2A6*	*CYP2B6*	*CYP2C9*	*CY2C19*	*CYP2D6*	*CYP2E1*	*CYP3A4*	*CYP3A5*
Deletion	47.6	0.4	27.2	0.4	0	0.4	2.0	0	0.4	0.4	1.6	0	0.4	0
Duplication	15.4	0	17.5	0	0	0	0.4	0.4	0.4	0	7.7	1.6	0	0.4
No CNV’s	37.0	99.6	55.3	99.6	100	99.6	97.6	99.6	99.2	99.6	90.7	98.4	99.6	99.6

## Discussion

The analysis of genomic variation in the general population is essential to understand the phenotypic diversity and its potential involvement in drug response. The Human Genome Project [11, 18], the SNP Consortium, The International Hap Map project [19], and more recently the 1000 Genomes Project and the Encyclopedia of DNA Elements (ENCODE) have collectively identified nearly 12 million SNP representing 26 populations around the world [20, 21]. Less is known on CNVs, even though they are suspected to be involved in genetic disease susceptibility and the efficacy/toxicity response to drugs [1, 9, 12, 22]. Due to current knowledge of CNVs in relation to drug efficacy and toxicity and the fact that its variation in Latin Americans is understudied, it’s necessary to conduct studies in these CNVs. In our study, the presence of CNVs were evaluated using the commercial kit SALSA MLPA P128-C1 Cytochrome P450 probe mix (#P128-C1, MRC-Holland, Amsterdam). At present, the SALSA MLPA P128-C1 Cytochrome P450 probe mix is the only available commercial analytical panel design for analyzing *Cytochromes P450* (*CYP*) genes deletions and duplications (https://www.mlpa.com) [23–25]. Genomic variants in human *CYPs* are a major source of variability in drug pharmacokinetics and response. CYP1, 2, and 3 families, are the principal metabolizing enzymes of phase I metabolism involved in most of medications [26–28]. Additionally, the panel includes *GST* genes related to detoxification of carcinogens, therapeutic chemicals and environmental toxins [29]. The accurate understanding of genomic variants prevalence related to drug toxicity and efficacy is important for proposing adequate therapeutic management.

We observed that 13 out of 14 genes studied (92.9%) presented deletions and/or duplications. Regarding these findings, the analysis of CNVs in 542 healthy unrelated individuals showed polymorphisms in 3 out of 11 *CYP-450* genes [6], while another study of CNVs found that *CYP1A1, CYP1B1*, and *CYP2B6* had no CNVs [29]. We consider that in the Colombian population there is greater variability in the *GST* and *CYP-450* genes than in other populations. Testing CNVs in GSTs and CYPs genes can allow the selection of patients for different starting dose regimes; indeed CNVs genotypes of these genes are predictors of response to treatment [24].

Our results indicated that nearly 50% of our population had one type of CNV (deletion or duplication) in one or several of the analyzed genes and 33% of the individuals had some genes with deletion and others with duplication. The individuals carrying nonactive alleles are classified as “predicted” poor metabolizers with no metabolic capacity for these enzymes. On the contrary, individuals with more than two active alleles have been associated with increased enzyme activity [14]. There are two risk scenarios in regards to the genetic background: the first is the administration of a prodrug that requires conversion to an active metabolite where slow metabolizers generate loss of efficacy. The second is the administration of a drug that is eliminated by a single pathway since its absence results in the accumulation of the parent drug generating toxicity. Coadministration of drugs that inhibit a bioactivating enzyme can result in “phenocopy” of the slow metabolizer phenotype [30]. Our study has revealed the existence of genotypic diversity that allowed the identification of 14 categories defined by the mutational status observed in the 14 genes included. 35% of the individuals were carriers of different combinations of CNVs that reflect the dynamics underlying this type of variant. Several mechanisms have been proposed in the generation of a CNV including non-allele homologous recombination, non-homologous end joining, fork stalling and template switching, and microhomology-mediated break-induced replication.

To date, findings show that CNV duplications are significantly more frequent than deletions [5, 31]; however, our study identified deletions as the predominant mutation. The allelic frequencies of deletions were greater in the *GST* than in the *CYP-450* genes: for *GSTM-1* the biggest value in the population was identified (50.4%) followed by *GSST-1* (35%), while for *CYP* the highest deletion allelic frequencies reported were 3.3% (*CYP2D6*) and 4.1% (*CYP2A6*). Regarding *GST* genes, their location among genomic regions of segmental duplications (SD) is relevant, since the regions flanked by SD are prone to rearrangement by nonallelic homologous recombination [32–34]. Our results are in concordance with others and indicate that deletions in *GST* genes are relatively common in different populations (23,7% to 51,6% for *GSTM1* and 4,25% to 46,8% for *GSTT1*) [12]. According to the function of *GSST1* and *GSTM1* in the detoxification of exogenous compounds, the individuals carrying deletions have an increased risk for several cancers (colorectal and chronic myeloid leukemia) and toxicities related to medications [35–39]. Some of the toxicity reactions are secondary to a combination of deletions in *GSST1* and *GSTM1* genes. Given the high prevalence of deletions in *GSST1* and *GSTM1*, 16.2% of the participants in our study were carriers of these double mutations, a finding that led to estimate the potential impact of these variants in our cohort of Colombian individuals.

The presence of deletions in other genes (*CYP-450* and *GSTP1*) was lower (0 to 1.6%) in relation to *GSST1* and *GSTM1*. Moreover, *CYP2D6* and *CYP2A6* presented an allele frequency greater than 1% with values of 1.6 and 2.0% respectively. With the exception of *CYP2D6*, little is known about the frequency of CNVs in these genes. The clinical and pharmacogenomic implication of *CYP2A6* deletion has been related to its role in the metabolism of nicotine, cotinine and nitrosamine, pre-carcinogens which increase the risk of tobacco-related cancer [40, 41]. In our study, allelic and genotypic frequencies for *CYP1A1* and *CYP1B1* null were 0.4 and 0.8% respectively. The genetic population characteristics of these CNVs are unknown. It has been estimated that the pharmacogenetic impact of CYP1A1 and CYP1B1 is lower compared to other family members of CYP-450, due to the fact that they are extrahepatic enzymes, and therefore have limited relevance in the elimination of substrates (caffeine, phenacetin, flunarizine, amiodarone and others). In accordance to other reports, our findings demonstrate an absence of CNVs in CYP1A2 [42], suggesting that *CYP1A2* is a conserved gene, for which common variants that significantly alter gene expression or enzyme activity have not been described [33]. Regarding the members of the *CYP2* family, allelic frequencies for *CYP2C9* and *CYP2C19* were identical (0.4%). Our findings, which are similar to those proposed by other authors, indicate that *CYP2C9* and *CYP2C19* duplications/deletions are rare in the population [43]. These findings suggest that the influence of CNVs in *CYP2C9* and *CYP2C19* in the pharmacological responses is less significant than SNVs.

*CYP2D6* metabolizes over 25% of drugs currently used in the clinical practice [26]. The deletion of the whole gene was present in our population with an allelic frequency of 1.6% in accordance with other admixed Americans populations (3%). Worldwide, there is ethnic variability with frequencies from 2 to 6.5%, which contribute essentially to the interindividual variability in the response to drugs observed in different populations [44]. The medical response of the *CYP2D6* deletion carriers has been widely documented and associated with the occurrence of ADRs generated by the high levels of parenteral drugs or by therapeutic failure secondary to the inability to create an active metabolite [45]. The duplications and multiduplications have been associated to *CYP2D6*, with individuals that carry among 2 to 13 gene copies. Our results indicated that the genotypic frequency of *CYP2D6*WT/*CYP2D6*Dup was 10.6%, while homozygotes for the polymorphism corresponded to 2.4%. The allelic frequency for duplications was 7.7%, greater than the reported by Zhou et al. (1%), who studied 5789 samples of admixed Americans [44]. It is possible that our population has its own profile in genes such as *CYP2D6*. Individuals with extra copies of *CYP2D6* correspond to the UM group in which each functional copy increases the metabolism rate of the enzyme substrate. The relationship between genotype and phenotype should be analyzed with caution since, although it has been established that heritability of interindividual differences of the drug response phenotype is near to 70%, the analysis of common variants has explained less than half of estimate heritability. Rare variants, different types of genomic variation and factors such as drug-drug interactions, are determinants in the multifactorial or complex behavior of the metabolic phenotype [46]. Recently, the emergence of “pharmacometabolomic-aided pharmacogenomics” reinforces the need to clinically identify and validate the potential associations of genetic, physiological, chemical and environmental influence related to the toxicity / efficacy of xenobiotics. This synergy can have a great impact in predicting the benefit of the therapeutic intervention in patients [47]. Some reports have established the need to analyze the clinical implication of pharmacogenetics from the genotypic, haplotype and phenotypic perspective and not only focus on one level of information, since genomic variants can vary throughout the different populations and their effect on the phenotype of interest can be modified by one or more variants [48]. For Latin American populations, including Colombia, it is common to see mixed populations with different percentages of ancestries (Table 5) and it is recognized that it is a continuous rather than a categorical variable, even within the self-reported race / color categories [49].

**Table 5:** Ancestry in different regions of Colombia [15]

		Type Marker	European (%)	African(%)	Amerindian(%)
Caribbean area	Santa Marta (n:26)	AIMs	50	28	22
	Cartagena (n:80)	AIMs	23	44	33
Northwest	Medellin (n:849)	AIMs	60	12	28
	Peque (n:163)	AIMs	32	6	62
	Manizales(n:203)	AIMs	59	4	37
	Bucaramanga(n:82)	AIMs	56	1	43
Central	Armenia(n:58)	AIMs	57	5	38
	Bogotá(n:24)	AIMs	45	3	52
	Boyacá(n:80)	SNPs	42	20	38
	Cundinamarca(n:19)	STRs	47	2	51
	Huila(n:82)	SNPs	41	19	40
Southwest	Pasto(n:201)	AIMs	32	3	65
	Popayán(n:61)	AIMs	20	23	57
	Neiva(n:24)	AIMs	39	0	61
Pacific coast	Quibdo(n:72)	AIMs	21	68	11

Our results highlight the variability and potential impact of *GST* and *CYP-450* genes in interindividual drug response. In terms of pharmacogenetic evaluation, we estimate that our results indicate that in the Colombian population there exists a significant allele frequency conferring susceptibility for an inadequate response to certain drugs; *GSTM1*, *GSTT1*, *CYP2D6*, and *CYP2A6* showed the greatest variability of CNVs. Duplications and deletions in *CYP2D6* (9.3% of the alleles identified) influence drug pharmacokinetics and subsequent pharmacological and toxicological effects [7].Those genomic variants impact about 25% of the drugs used clinically (e.g. amiodarone, amitriptyline, clomipramine, codeine, tramadol, fluoxetine, simvastatin) in therapeutic areas related to psychiatry, cardiology, and oncology [28]. The analysis of CNVs for *CYP2D6* has been documented in clinical management guidelines established by international consortiums such as the Dutch Pharmacogenetics Working Group guidelines (DPWG), and the Clinical Pharmacogenetics Implementation Consortium (CPIC). Clinical evidence has suggested that *CYP2D6* genetic testing provide useful information to guide drug dosage and interpretation of potential patients’ metabolizer phenotypes. Concerning the *GST* genes, the individuals carrying deleted alleles (e.g. *GSTT1* and *GSTM1* null) are of special interest regarding the response to antineoplastic agents for cancer treatment. Interestingly, since *CYP2A6* variants have been related to antiretroviral therapy our results might be useful for delineate therapeutic strategy accurately in Colombian HIV/AIDS patients. Until now, although some important evidence regarding the impact of CNVs on the toxicity and efficacy of drug response has been published, the translation of this knowledge into clinical practice has not been widely determined. The incorporation of CNVs genetic testing into health system is therefore still uncertain.

Taking together, our results allow us to establish for the first time a profile of CNVs for GST and CYP-450 genes in a cohort of Colombian individuals. We estimate that our results are representative for the Colombian and Latin American population with ancestry (reported in the literature by AIMs) similar to that attributed to the healthy people evaluated in this work (Table 5).

We consider that the principal limitation is the non-detection of copy number changes that lie outside the target sequences of probes incorporated in the SALPA MLPA P128-C1 Cytochrome P450 probe mix. In the case of *CYP2D6*, MLPA does not allow the discrimination of the presence of duplications in active genes, which would require additional analysis capable of identifying CNVs and SNVs simultaneously. Additionally, our study lacks an ancestry analysis of the participants; therefore genetic background cannot be established with accurately.

## Conclusion

Our results describe the first genomic profile of CNVs for *GST* and *CYP* genes in a cohort of the Colombian population. These findings are relevant due to the impact of these genes in the pharmacogenomic drug selection and dosing, adverse drug reactions and disease susceptibility. Additionally, our search serves to the understanding of the CNVs frequency and potential health impact, so far unknown in other Latin American populations.

## Data Availability

The software of MLPA analysis used during the current study is available at https://www.mlpa.com/WebForms/WebFormMain.aspx. Data obtained in our study is available from the corresponding author on request.

## Abbreviations

ADME: Drug absorption, distribution, metabolism and excretion
ADRs: Adverse drug reactions
AIMs: Ancestry informative marker sets
CNVs: Copy number variations
EMA: The European Medicines Agency
ENCODE: The Encyclopedia of DNA Elements
FDA: US Food and Drug Administration
MLPA: Multiplex ligation-dependent probe amplification
PCR: Polymerase chain reaction
PM: Poor metabolizer
SD: Segmental duplications
SNVs: Simple nucleotide variants
UM: Ultrarapid metabolizers
